# Oncologic control and predictors of urologic reconstruction after Mohs micrographic surgery for low-risk penile malignancy

**DOI:** 10.1007/s11255-024-04121-6

**Published:** 2024-06-26

**Authors:** Brian D. Cortese, Raju Chelluri, Alexander J. Skokan, Leilei Xia, David A. Ostrowski, Daniel S. Roberson, Lauren Schwartz, Daniel J. Lee, Tess M. Lukowiak, Thomas J. Guzzo, S. Bruce Malkowicz, Christopher J. Miller, R. Caleb Kovell

**Affiliations:** 1 Perelman School of Medicine, University of Pennsylvania, Philadelphia, PA USA; 2 Department of Urology, Fox Chase Cancer Center, Philadelphia, PA USA; 3 Department of Urology, University of Washington, Seattle, WA USA; 4 University of Southern California Institute of Urology, Keck School of Medicine, University of Southern California, Los Angeles, CA USA; 5 Department of Surgery, Division of Urology, University of Pennsylvania Health System, 800 Walnut Street, Urology Offices, Philadelphia, PA USA; 6 Department of Pathology, University of Pennsylvania Health System, Philadelphia, PA 19107 USA; 7 Department of Dermatology, Rutgers Robert Wood Johnson, Somerset, NJ USA; 8 Department of Dermatology, University of Pennsylvania Health System, Philadelphia, PA USA

**Keywords:** Penile neoplasms, Mohs surgery, Reconstruction, Oncologic outcomes, Neoplasm recurrence

## Abstract

**Purpose:**

Mohs micrographic surgery (MMS) is a low-risk penile cancer management option. However, contemporary patients’ short-term oncologic control and preoperative characteristics predicting reconstruction needs are undefined. This study assesses MMS’s oncologic efficacy for low-risk penile cancer and identifies baseline predictors of post-resection reconstruction referral.

**Methods:**

We retrospectively reviewed 73 adult males with 78 penile cutaneous malignancies treated with MMS from 2005 to 2019. Patients underwent MMS with or without surgical reconstruction. Demographic information, MMS operative details, lesion pathology, and short-term outcomes were recorded. Descriptive statistics for all variables were calculated, and logistic regression identified predictive factors for urologic referral for complex reconstruction.

**Results:**

Seventy-three men with 78 lesions, all staged ≤ cT1a prior to MMS, were identified. Twenty-one men were found to have invasive SCC. Median follow-up was 2.0 years (IQR 0.8–5.2 years). MMS was able to clear the disease in 90.4% of cases. One patient had disease related death following progression. Dermatology closed primarily in 68% of patients. Twenty percent of patients had a complication, most commonly poor wound healing. On univariate and multivariate linear regression analysis, lesion size > 3 cm and involvement of the glans independently predicted the need for referral to a reconstructive surgeon.

**Conclusions:**

MMS for penile cancer appears to provide sound oncologic control in the properly selected patient. Involvement of a reconstructive surgeon may be needed for glandular and large lesions, necessitating early referral to a comprehensive multidisciplinary care team.

## Introduction

Penile cancer (PC) is an uncommon malignancy [1] generally treated with partial or total penectomy, potentially followed by inguinal lymph node dissection (ILD) [2]. Low-risk lesions (PeIN, Tis, Ta, and T1a) may be treated with less aggressive approaches, including topical therapy or wide local excision [3]. Mohs micrographic surgery (MMS) has been used for low-risk PC management [2]. A 2007 series reported 1/3 of PC patients had recurrence following resection with MMS [4], but proper patient selection may yield lower recurrence rates. Furthermore, some patients undergoing MMS for PC will require referral for surgical reconstruction. Pre-operative anticipation for referral may improve care coordination and patient counseling.

Contemporary literature describing oncologic outcomes after MMS for PC as well as factors predicting genitourinary (GU) reconstruction are limited. This study aims to determine the oncologic efficacy of MMS when used in managing low-risk, localized PC patients. A further goal was to identify baseline predictors of referral for post-resection reconstruction. We hypothesized that MMS could provide effective oncologic control for low-risk, localized PC in carefully selected patients.

## Patients and methods

**Fig. 1:**
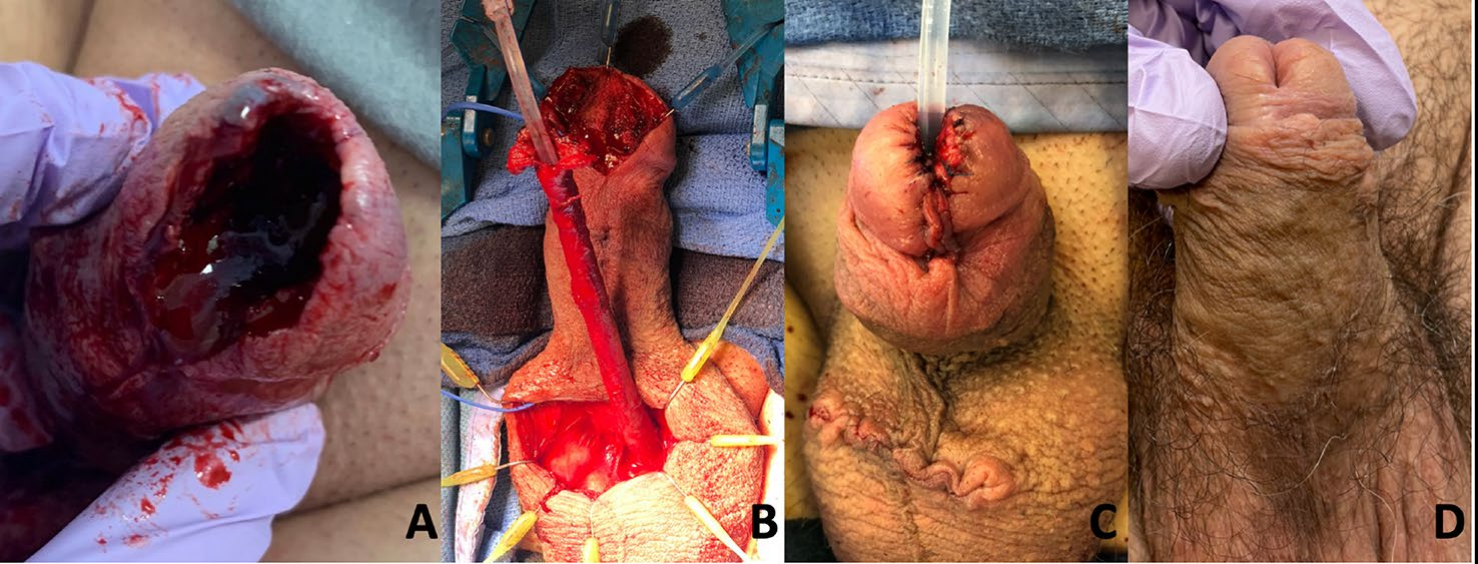
Images of a penile cancer patient in our cohort depicting a large surgical defect of the glans including the meatus and distal urethra following MMS (**A**), intraoperative image from reconstructive procedure showing a mobilized distal urethra (**B**), penis immediately following reconstructive procedure (**C**), and short term follow up of reconstructed penis (**D**)

A retrospective analysis was conducted on a prospectively maintained institutional database covering patients who underwent GU MMS from 2005 to 2019 (n = 101). The COVID-19 pandemic interrupted our service line and data collection. The Institutional Review Board approved the exclusion of informed consent due to the study’s deidentified dataset and retrospective design. The technical aspects of Mohs micrographic surgery have been detailed extensively in the literature [5, 6]. Figure 1 Illustrates an example of the pre- and post-MMS procedure and reconstruction. In this cohort, MMS was performed by five dermatologists trained in MMS, with the majority (~ 90%) conducted by two providers. Final pathology was reviewed by either of two GU-specialized pathologists.

For each MMS case, patient-level data was deidentified and recorded in a prospectively maintained database. A chart review for patient follow-up and on a per-lesion basis was conducted using operative reports. If available, collected data included: ethnicity, occupation, age, duration of follow-up, risk-altering health status (immunosuppression, HIV, HPV, circumcision, smoking status), survival, cause of death, pre- and post-MMS diagnosis, pathologic subtype, original diagnosis date, any original pathology or prior treatment, location, pre- and post-operative lesion size, number of stages, final margin status, final pathologic stage, type of closure, post-MMS urologic referral, requirement of additional oncologic or reconstructive surgeries, reconstruction type, clinically significant complications, recurrence from index MMS procedure, and date/pathology/time to recurrence. Follow-up was reviewed until each patient’s last documented visit with Urology or Dermatology.

Data was captured in Excel v.16 (Microsoft; Seattle, Washington). Statistical analysis was performed with STATA SE 15.0 (Statacorp, LLC; College Station, Texas). For continuous variables median with interquartile range (IQR) are reported with means when appropriate. Univariate and multivariate logistic regression analysis was performed to identify predictors for need of formal reconstruction. Covariates were chosen based on ease of clinical ascertainment and thus easily fit into the clinical evaluation and workflow. Statistical significance was defined as p < 0.05.

## Results

A total of 101 patients who underwent MMS of GU lesions at our institution were identified. Seventy-three men, with a median age of 64 years, underwent resection of 78 penile tumors (Table 1). All patients with penile lesions had been biopsied by an outside provider before referral for MMS. Initial biopsies revealed Bowenoid papulosis in 2 patients, PeIN/CIS in 55 patients, and T1a squamous cell carcinoma (SCC) in 16 patients. The median lesion size (largest dimension) was 2.4 cm (IQR 1.45–3.55 cm), with most lesions found on the penile shaft. All lesions diagnosed as SCC before referral were staged ≤ cT1a prior to MMS. In the total cohort, 21.9% (16/73 cases) were immunosuppressed, 24.7% (18/73 cases) were uncircumcised, and 9.6% (7/73 cases) received topical treatment before referral, of which 4 patients received imiquimod and 3 patients received topical cream or ointment not otherwise specified. The median follow-up was 2.0 years (IQR 0.8–5.2 years). The final SCC cohort comprised 21 men. Of these men, 23.8% (5/21) were initially diagnosed as PeIN/CIS on preprocedural biopsy; the post-MMS procedural pathology revealed more extensive disease with four cases showing pT1 and one case showing pT2.

**Table 1:** Patient and tumor characteristics

	N (%) or mean/median (IQR)		
	Total	Invasive SCC	CIS
*Patient characteristics*			
Number of patients	73 (100%)	21 (28.8%)	52 (71.2%)
Age (years)	60/64 (50 – 70)	64/66 (56–77)	59/60 (50–68)
Ethnicity			
Caucasian	52 (71.2%)	11 (52.4%)	41 (78.8%)
African American	14 (19.2%)	6 (28.6%)	8 (15.4%)
Hispanic	3 (4.1%)	2 (9.5%)	1 (1.9%)
Asian American	2 (2.7%)	2 (9.5%)	0
Other/unknown	2 (2.7%)	0	2 (3.8%)
Immunosuppressed or Immunocompromised	16 (21.9%)	5 (23.8%)	11 (21.2%)
HIV/AIDS	7 (9.6%)	2 (9.5%)	5 (9.6%)
Organ transplant	9 (12.3%)	3 (14.3%)	6 (11.5%)
HPV positive	13 (17.8%)	3 (14.3%)	10 (19.2%)
Circumcised	55 (75.3%)	13 (61.9%)	42 (80.8%)
Smoking status			
Never	25 (34.2%)	9 (42.9%)	16 (30.8%)
Former	27 (37.0%)	8 (38.1%)	19 (36.5%)
Current	16 (21.9%)	4 (19.0%)	12 (23.1%)
Unknown	5 (6.8%)	5 (23.8%)	0
*Tumor characteristics*			
Recurrence of prior local disease	6 (8.2%)	2 (9.5%)*	4 (7.7%)**
Locations involved			
Glans	24 (32.9%)	16 (76.2%)	8 (15.4%)
Prepuce	11 (15.1%)	4 (19.0%)	7 (13.5%)
Shaft	48 (65.8%)	10 (47.6%)	38 (73.1%)
Preoperative biopsy pathology			
Invasive SCC	16 (21.9%)		0
CIS	55 (75.3%)	16 (76.2%)	50 (96.2%)
Indeterminate	2 (2.7%)	5 (23.8%)	2 (3.8%)

*Prior T1a disease, treated previously with laser ablation in both cases

**Prior CIS, treated with laser ablation, excision, or topical imiquimod

For patients diagnosed with SCC, approximately 29% (6/21 cases) of initially T1a lesions demonstrated risk upgrading (Table 2). This includes 23.8% of cases unable to be cleared by MMS (5/21 cases), 19.0% with upstaging to pT1b (4/21 cases), and 4.8% with upstaging to pT3 disease (1/21 cases). One man had residual disease at the urethral margin requiring distal urethrectomy. Another patient with pT1bNx SCC of size 4 cm × 2.5 cm on their glans underwent a partial penectomy with negative margins, but subsequently had a recurrence 8–9 months later with cN1 disease in the left superficial inguinal lymph node.

**Table 2:** Pathologic and clinical outcomes

	N (%) or mean/median		
	Total	Invasive SCC	CIS
Final pathology			
CIS	52 (71.2%)	0	52 (100%)
T1a	15 (20.5%)	15 (71.4%)	0
T1b	4 (5.5%)	4 (19.0%)	0
T2	0	0	0
T3	1 (1.4%)	1 (4.8%)	0
Failure of MMS			
(+) Carcinoma	3 (4.1%)	3 (14.3%)	0
(+) CIS	3 (4.1%)	1 (4.8%)	2 (3.8%)
Dysplasia	1 (1.4%)	1 (4.8%)	0
Complications (after MMS)			
Bleeding/hematoma	2 (2.7%)	0	2 (3.8%)
Infection	1 (1.4%)	0	1 (1.9%)
Poor wound healing	9 (12.3%)	3 (14.3%)	6 (11.5%)
Meatal stenosis	2 (2.7%)	1 (4.8%)	1 (1.9%)
Other	1 (1.4%)	0	1 (1.9%)
Local recurrence	1 (1.4%)	0	1 (1.9%)
Disease progression			
Invasive (from CIS)	0	0	0
Nodal	1 (1.4%)	1 (4.8%)*	0
Metastatic	1 (1.4%)	1 (4.8%)*	0
Death			
Disease-related	1 (1.4%)	1 (4.8%)*	0
Unrelated	3 (4.1%)	1 (4.8%)	2 (3.8%)

*T1b pathology treated with radical resection after Mohs, with both patients subsequently requiring nodal dissection

Conversely, for patients initially diagnosed with CIS, approximately 3.8% (2/52 cases) of CIS lesions experienced failure of MMS to clear the lesion. One patient had a final margin read as dysplasia, without recurrence to date. Another man had residual CIS of his meatus, but cystourethroscopy and biopsy yielded benign mucosa. Two others had residual CIS at the urethral meatus margin managed with distal urethrectomies. Lastly, one man was downstaged from PeIN/CIS to Bowenoid papulosis and two men had only benign findings from their MMS procedure (both had PeIN/CIS diagnoses prior to MMS).

In aggregate, sixty-six men, ~ 90% of the total cohort, achieved clear margins by MMS. Notably, the last failure patient, early in our series, was referred for total penectomy which was performed at an outside institution. All other patients received their completion resection at our institution.

Table 2 illustrates the recurrence, progression, and survival outcomes in our cohort. One of 21 (4.8%) patients with SCC developed nodal disease requiring an ILD. This patient, described earlier, developed metastatic disease following ILD, and is our only patient in this program to date to have a PC-related death. One of 52 (1.9%) patients diagnosed with CIS had a local recurrence, managed by repeat MMS. Three other men (one with SCC and two with CIS) died of causes unrelated to their disease.

Sixty-eight percent of patients were closed primarily by the dermatologist, with the rest requiring reconstruction by plastic surgery or reconstructive urology. Primary closure by a plastic surgeon was performed in one patient. Of the twenty-two patients treated by a reconstructive surgeon, five (22.7%) underwent closure with a skin graft (split or full thickness), nine (40.9%) were closed with local tissue flaps or tissue rearrangement, and five (22.7%) underwent a combination of both techniques. One patient required meatoplasty. One patient required urethroplasty with glansplasty. Twenty percent (15/73 cases) of patients had a complication from surgical reconstruction including meatal stenosis, scarring, graft loss, hematoma, wound dehiscence, and inclusion cysts (Table 2).

On univariate and multivariate (Table 3) analysis, the two chosen measurable, preoperative factors that were associated with need for reconstruction were: (1) lesions 3 cm or larger in greatest dimension at the time of initial measurement (HR 9.83, 95% CI 2.81–34.4, p < 0.0001) and (2) involvement of the glans (HR 5.54, 95% CI 1.58–19.4, p = 0.008).

**Table 3:** Multivariate logistic regression for predictive factors of reconstructive surgical referral after Mohs micrographic surgery for penile cancer

Clinical paranmeter	Univariate		Multivariate	
	Hazard ratio (95% CI)	P value	Hazard ratio (95% CI)	P value
Glans involvement	11.2 (3.63–33.1)	< 0.001	9.83 (2.81–34.4)	< 0.0001
Lesion size over 3 cm	5.83 (1.86–15.5)	0.0013	5.54 (1.58–19.4)	0.008

## Discussion

Our study reports the efficacy of MMS at our institution for patients with low-risk penile lesions (initially ≤ cT1a). MMS was applied to primarily low-grade lesions on the penile shaft, achieving successful clearance in about 90% of cases and failing in 23.8% of SCC and 3.8% of CIS. Disease progression occurred in about 9.5% of the SCC cohort, with a local recurrence rate of 1.9% in the CIS cohort. One patient with SCC died of PC. Overall, 20% experienced surgical complications, all classified as Clavien-Dindo grade II or below, [7–11] and 30% underwent surgical closure by a reconstructive urologist or plastic surgeon. Glans involvement (HR 5.54, 95% CI 1.58–19.4, p = 0.008) and lesion size > 3 cm (HR 9.83, 95% CI 2.81–34.4, p < 0.0001) predicted involvement by a reconstructive surgeon on multivariate analysis, indicating that patients with these preoperative risk factors are significantly more likely to require complex surgical intervention and highlighting the need for early identification and potentially more aggressive treatment planning to improve patient outcomes.

The historical efficacy of MMS for managing penile SCC is limited. In 1985, Mohs published a series of 29 penile SCC patients (25 determinate cases) with a five-year cure rate of 68% [12]. Brown et. al published a series two years later describing 20 patients; four developed metastatic disease to the lymph nodes and one died of metastatic penile SCC [13]. Shindel et. al reported 33 patients who received MMS from 1988 to 2006, of which 32% had recurrence and two had disease progression leading to death due to PC [4]. Additionally, they reported a ~ 27% complication rate (9/33 patients), comparable to our series with a reported complication rate of 20% [4]. In 2016, Machan et al., described their 30-year series of MMS for penile SCC, with 44 patients nearly split in half between PeIN/CIS and SCC [14]. They reported a 94.7% cure rate for PeIN/CIS, with one recurrence cleared with MMS [14]. Our data demonstrates a 1.4% local recurrence rate, but we recognize that the PeIN/CIS and SCC cohorts are not identical based on disease staging in these retrospective series.

Local referral patterns can significantly impact initial management, especially when patients are diagnosed with penile SCC or PeIN/CIS without urologic consultation. Urologist referral allows for a combined approach to management and completing clinical staging with guidelines-based surveillance for early disease recurrence. In our study, almost all patients with disease upgrading were appropriately referred to a urologist for further management. Additionally, approximately one third of patients treated with MMS had soft tissue defects requiring advanced reconstructive techniques involving flaps, grafts, and local tissue rearrangements to achieve satisfactory cosmetic and functional outcomes.

Low-risk PC has several management options [3], including topical therapy with 5-fluorouracil or imiquimod, wide local excision, or laser ablation (CO2, Nd:YAG, and KTP) [15]. In 2012, Alnajjar et al. investigated the efficacy of topical agents in patients with PeIN/CIS [16]. In 44 patients, approximately 50% of those treated topically showed complete resolution after a mean of 34 months follow-up [16]. Further, their institution’s protocol would only offer follow-up for lesions 1–2 cm in size, while our median lesion size pre-MMS was ~ 2.5 cm. In 2018, Sri et al. published their series of 322 men undergoing wide local excision for penile SCC leading to a 4% recurrence rate, although PeIN/CIS was excluded [17]. Laser ablation efficacy depends on laser type and lesion pathology. In 2017, Leone et al. reported CO2 laser recurrence rates were ~ 15 to 25%, while Nd:YAG recurrence rates were ~ 10 to 50% in men with PeIN/CIS and T1 penile SCC [18].

This study has several limitations. This retrospective analysis is hypothesis-generating and introduces selection bias. As a single center, our results and referral patterns may lack generalizability. The median follow-up was relatively short, and the cohort is small, consisting of approximately 5 cases annually over 14 years. Furthermore, our risk prediction model is limited intentionally with a goal of clinical applicability in mind. Additionally, we do not compare our results to non-MMS techniques such as total glans resurface. However, this is the largest cohort describing MMS resection of PC. Also, we report on referral patterns and involvement of secondary reconstructive surgeons following MMS for PC, an area of paucity in the literature.

Our data suggest that a multidisciplinary approach may be suitable for many low-risk PC cases. MMS could potentially optimize penile tissue for cosmetic and functional outcomes in carefully selected patients. Further prospective studies could determine the efficacy of MMS and the patient population best served by this technique, especially considering the high risk of recurrence, as well as investigating the impact of same-session reconstruction on wound healing outcomes. Defining eligibility for MMS is a crucial step for future developments.

## Conclusions

In carefully selected patient, MMS can treat low-risk PC with safe oncologic outcomes for CIS, but additional data are required to conclude the same for SCC. While most patients can be closed primarily during MMS, those with large lesions (> 3 cm) and glans involvement may require reconstructive surgery. Recognizing early predictive clinical signs may facilitate treatment by a comprehensive multidisciplinary care team.

## Data Availability

No datasets were generated or analysed during the current study.
